# Neurotrophic factors in Alzheimer’s disease: role of axonal transport

**DOI:** 10.1111/j.1601-183X.2007.00378.x

**Published:** 2008-02

**Authors:** K Schindowski, K Belarbi, L Buée

**Affiliations:** † Institut National de la Santé et de la Research Médicale (INSERM) U837 Lille Cedex, France; ‡ Faculté de Médecine, Institut de Médecine Prédictive et Recherche Thérapeutique, Université Lille 2 Lille Cedex, France

**Keywords:** Abeta, APP, BDNF, cholinergic neurons, dementia, neurodegeneration, NGF, NT-3, NT-4/5, Tau

## Abstract

Neurotrophic factors (NTF) are small, versatile proteins that maintain survival and function to specific neuronal populations. In general, the axonal transport of NTF is important as not all of them are synthesized at the site of its action. Nerve growth factor (NGF), for instance, is produced in the neocortex and the hippocampus and then retrogradely transported to the cholinergic neurons of the basal forebrain. Neurodegenerative dementias like Alzheimer’s disease (AD) are linked to deficits in axonal transport. Furthermore, they are also associated with imbalanced distribution and dysregulation of NTF. In particular, brain-derived neurotrophic factor (BDNF) plays a crucial role in cognition, learning and memory formation by modulating synaptic plasticity and is, therefore, a critical molecule in dementia and neurodegenerative diseases. Here, we review the changes of NTF expression and distribution (NGF, BDNF, neurotrophin-3, neurotrophin-4/5 and fibroblast growth factor-2) and their receptors [tropomyosin-related kinase (Trk)A, TrkB, TrkC and p75^NTR^] in AD and AD models. In addition, we focus on the interaction with neuropathological hallmarks Tau/neurofibrillary tangle and amyloid-β (Abeta)/amyloid plaque pathology and their influence on axonal transport processes in order to unify AD-specific cholinergic degeneration and Tau and Abeta misfolding through NTF pathophysiology.

## From ‘healthy’ aging to Alzheimer’s disease

Alzheimer’s disease (AD) is a neurodegenerative disorder that is characterized by global cognitive decline including a progressive loss of memory, orientation and reasoning. The neurologist and psychiatrist Alois Alzheimer extensively described a dementia syndrome of his patient D. Auguste, whom he treated in Frankfurt am Main, Germany at the beginning of the past century (Jarvik & Greenson 1987). He recorded a rapidly progressing memory loss of the 52-year-old woman. After her death, he examined her brain and found histological changes that are specific for AD.

Age-associated dementias like AD are becoming more and more important in industrialized countries as life expectancy increased by 2 years per decade during the recent 20 years (Klenk *et al.*2007). The incidence of age-associated dementias is about 1.3% of the total population of Western Europe; among them, AD is the most common, affecting 50% of all demented patients (Ferri *et al.*2005; Hofman *et al.*1991). This is likely to increase dramatically in the next 35 years. According to recent estimations, the number of people with dementia over the age of 60 will be approximately doubled in 2040. An irreversible loss of cognitive and mental abilities is the prognosis of this disorder. In later stages, demented patients are helpless and require full-time nursing care. Besides the personal and familial tragedies that are an aspect of dementia, AD and other dementias are a financial problem for the health service and, thereby, a burden for the whole social community. And this cost will rise in future as more and more persons are aging and becoming older.

## Neuropathological changes in the AD brain

Histologically, the neurodegeneration is distinguished by neuropathological changes and deposits of misfolded proteins, mainly consisting of hyperphosphorylated Tau in neurofibrillary tangles and amyloid-β (Abeta) in the form of senile plaques and deposits in cerebral blood vessels.

### Neurofibrillary tangles

Neurofibrillary tangles consist of hyperphosphorylated Tau proteins that aggregate inside neurons along neurites – observed as neuropil threads – and finally in the soma. Tau proteins belong to the microtubule-associated protein family. They are mainly found in neurons. Nonneuronal cells usually display trace amounts, but in some diseases, accumulation of tau in glial cells is detected (Bergeron *et al.*1997).

The human Tau gene is located on chromosome 17 and contains 16 exons. Alternative splicing of three of these exons (exons 2, 3 and 10) allows for six combinations (2−3−10−; 2+3−10−; 2+3+10−; 2−3−10+; 2+3−10+ and 2+3+10+) in the human brain. Tau proteins constitute a family of six isoforms, which range from 352 to 441 amino acids and have a high number of phosphorylation sites. Tau proteins bind microtubules through repetitive regions in their C-terminal part. These repetitive regions are the repeat domains (R1–R4) encoded by exons 9–12. The three (3R) or four copies (4R) are made of a highly conserved 18-amino acid repeat separated from each other by less conserved 13- or 14-amino acid interrepeat domains. Furthermore, the six Tau isoforms appear not to be equally expressed in neurons (for detailed review, see Sergeant *et al.*2005). Tau proteins are known to act as promoters of tubulin polymerization *in vitro*and are involved in axonal transport.

A couple of evidences support a role for the microtubule-binding domain in the modulation of the phosphorylation state of Tau proteins. In a low phosphorylated state, Tau binds to microtubules through the microtubule-binding domains and stabilizes their polymerization and assembly. However, microtubule assembly depends partially upon the phosphorylation state as phosphorylated Tau proteins are less effective than nonphosphorylated Tau proteins on microtubule polymerization. Phosphorylation inside and outside the microtubule-binding domains can strongly influence tubulin assembly by modifying the affinity between Tau and microtubules. However, properly assembled microtubules are essential to maintain axonal transport processes.

Most of the kinases involved in Tau phosphorylation include mitogen-activated protein kinase (MAPK), Tau-tubulin kinase and cyclin-dependent kinase. Stress-activated protein kinases have also been recently linked to Tau phosphorylation. Glycogen synthase kinase-3β (GSK-3β) is a Tau kinase that is able to phosphorylate both non-Ser/Thr-Pro sites and Ser/Thr-Pro sites.

In numerous neurodegenerative disorders, Tau proteins aggregate into intraneuronal filamentous inclusions. In AD, these filaments are named paired helical filaments (PHF).

Few phosphorylation-dependent antibodies such as AT100, AP422 or TG3/MC1 antibodies only detect PHF-tau, demonstrating the presence of abnormal phosphorylated sites. With the exception of Ser422, these phosphorylated sites found in PHF-tau are in addition conformation-dependent epitopes (Sergeant *et al.*2005). There is a direct relationship between hyperphosphorylation, abnormal phosphorylation and Tau aggregation, but it remains to be determined whether phosphorylation is a cause or a consequence in the aggregation process.

During normal aging, Tau hyperphosphorylation occurs in the transentorhinal cortex and spreads from here through the entorhinal cortex to the hippocampus (Braak & Braak 1991; Delacourte *et al.*2002). Once the hippocampus is reached, amyloid plaques may occur, and then the Tau pathology spreads over to the basal forebrain and several cortical areas in a distinct pattern along neuronal projections. Only the coexistence of Tau and amyloid pathologies is determined as AD.

To comprehend the role and mechanism of Tau pathology in AD, it is important to understand the normal function and processing of the Tau protein and the abnormal posttranslational processing of Tau in tauopathies. Mutations in the Tau gene have been found in several non-AD tauopathies and autosomal-dominant neurodegenerative disorders that exhibit extensive neurofibrillary pathology. However, Tau pathology observed in aging and AD is sporadic and not related to any mutation.

### Amyloid plaques

A major feature of both sporadic and familial forms of AD is the accumulation and deposition of Abeta – a peptide of 39–43 residues – within the brain tissue of AD sufferers. The accumulation of Abeta is thought to play a pivotal role in neuronal loss or dysfunction through a cascade of events that include the generation of free radicals, mitochondrial oxidative damage and inflammatory processes. The primary event that results in the abnormal accumulation of Abeta is thought to be the dysregulated proteolytic processing of its parent molecule, the amyloid precursor protein (APP) located on chromosome 21 (Selkoe 2001). The APP molecule is a transmembrane glycoprotein that is proteolytically processed by two competing pathways, the nonamyloidogenic and the amyloidogenic (Abeta-forming) pathways. How these pathways are regulated remain unclear. Three major secretases are postulated to be involved in the proteolytic cleavage of APP. These include α-secretase (of which the metalloproteases a disintegrin and metalloprotease (ADAM)17/TNF-alpha converting enzyme (TACE) and ADAM10 are likely candidates), beta APP cleaving enzyme (BACE, formally known as β-secretase) and the γ-secretase. The α-secretase cleaves within the Abeta domain of APP, thus precluding the formation of Abeta and generating nonamyloidogenic fragments and a secreted form of APP (α-APPs). In the amyloidogenic pathway, BACE cleaves near the N-terminus of the Abeta domain on the APP molecule, liberating another soluble form of APP, β-APP, and a C-terminal fragment (C99) containing the whole Abeta domain. The last step in the amyloidogenic pathway is the intramembranous cleavage of the C99 fragment by γ-secretase, to liberate a number of Abeta isoforms of 39- to 43-amino acid residues in length (Verdile *et al*. 2004). The same γ-secretase complex that generates Abeta may also generate the APP intracellular domain. The most common isoforms are Abeta_40_ and Abeta_42_; the shorter form is typically produced by cleavage that occurs in the endoplasmic reticulum, while the longer form is produced by cleavage in the trans-Golgi network. The Abeta_40_ form is the more common of the two, but Abeta_42_ is the more fibrillogenic because of its more hydrophobic nature and is, thus, associated with disease states. The γ-secretase enzyme is thought to be an aspartyl protease that has the unusual ability to regulate intramembrane proteolysis (for review, see Wolfe & Kopan 2004). The mechanism of γ-secretase activity is not yet known. Four components of the γ-secretase complex, presenilins, nicastrin, anterior pharynx defective (aph-1) and presenilin enhancer 2 (pen-2), have been identified.

Recently, it was shown that Abeta_42_ aggregates into oligomers within endosomal vesicles and along microtubules of neuronal processes, in cultured neurons, in APP transgenic mice and in human AD brain (Takahashi *et al.*2004). The oligomers that form on the amyloid pathway may be the cytotoxic species rather than the mature fibrils (Kayed *et al.*2003). Subsequently, anterograde axonal transport delivers Abeta to plaques (Lazarov *et al.*2002; Stokin *et al.*2005).

The sites of APP processing and Abeta release have yet remained unclear. Some studies speculate that the axon is the site of Abeta production (Muresan and Muresan, 2006). According to this, amyloid deposition would increase if poor axonal transport delays the progress of APP and its processing enzymes through the axon (Stokin *et al.*2005) but decreases when overexpression of BACE shifts Abeta generation away from the axon and synapse into the cell soma (Lee *et al.*2005a). But not all reports can reproduce part of this model, in which APP is cotransported with its processing enzymes (Goldsbury *et al.*2006; Lazarov *et al.*2005). Some Abeta release occurs at synapses (Lazarov *et al.*2005; Sheng *et al.*2002) and appears to be dependent on synaptic activity (Cirrito *et al.*2005). However, the occurrence of plaques in white matter tracts that lack synaptic input and the release of Abeta in primary neuronal cultures that lack synapses suggest that Abeta might be released from more proximal sites too (Qiu *et al.*2001; Wirths *et al.*2007). Indeed, if all Abeta release were at presynaptic endings, impairing axonal transport should decrease amyloid deposition instead of increasing it.

Autosomal-dominant mutations in APP cause hereditary early-onset AD, likely as a result of altered proteolytic processing. Increase in either the total Abeta levels or the relative concentrations of both Abeta_40_ and Abeta_42_ has been implicated in the pathogenesis of both familial and sporadic AD (Lue *et al.*1999).

### Three hypotheses for the pathogenesis of AD

The underlying molecular mechanisms of AD pathogenesis have not yet been identified; therefore, three major hypotheses have been advanced regarding the primary cause. The earliest hypothesis suggests that deficiency in cholinergic signaling initiates the progression of the disease. Two alternative misfolding hypotheses instead propose that either Tau protein or Abeta initiates the cascade.

The oldest hypothesis is the ‘cholinergic hypothesis’. A particular hallmark of AD is the specific neurodegeneration of cholinergic neurons leading to a loss of the neurotransmitter acetylcholine (ACh). Loss of cholinergic neurons seems to be specifically associated with typical clinical symptoms, like memory deficits, impaired attention, cognitive decline and reduced learning abilities (Hasselmo & Stern 2006; Kar *et al.*2004). All the first-generation therapeutics against AD were based on this hypothesis and work to preserve ACh by inhibiting its degrading enzyme acetylcholine esterase (AChE). These medications have not led to a cure. In all cases, they have served to only treat symptoms of the disease and can delay the progression of AD by 1–2 years but failed to reverse it. Therefore, it was concluded that ACh deficiencies may not be directly causal. More recently, cholinergic effects have been proposed as a potential causative agent for the formation of plaques and tangles (Shen 2004).

Later theories center on the effects of the misfolded and aggregated proteins Tau and Abeta. The hypothesis that Tau is the primary causative factor has been grounded on the fact that AD neuropathology starts in most individuals with hyperphosphorylated Tau and neurofibrillary tangles long before the first signs of Abeta occur (Braak & Braak 1991; Delacourte *et al.*2002). Nevertheless, accumulations of amyloid are frequently found in the cortex of nondemented individuals in the absence of neurofibrillary changes. A mechanism for neurotoxicity could be that hyperphosphorylated and aggregated Tau impairs axonal transport in murine Tau transgenic models (Ishihara *et al.*1999; Lewis *et al.*2000; Probst *et al.*2000), invertebrate models (Chee *et al.*2005; Kraemer *et al.*2003; Mudher *et al.*2004) and cellular models (Mandelkow *et al.*2004; Seitz *et al.*2002; Stamer *et al.*2002). Problems with axonal transport are believed to be a major cause leading to the symptoms and pathology observed in AD and other neurodegenerative dementias (Adalbert *et al.*2007). However, up to now, the preexistence of Tau pathology before the occurrence of Abeta pathology has not been shown in any experimental Tau model.

Abeta protein is a key molecule in the pathogenesis of AD. The tendency of Abeta to aggregate, its reported neurotoxicity and genetic linkage studies has led to the amyloid cascade hypothesis (Hardy & Allsop 1991). In this hypothesis, an increased production of Abeta results in neurodegeneration and ultimately dementia through a cascade of events (Verdile *et al.*2004). Amyloidogenic mouse models have established that overproduction of Abeta leads to dystrophic axons and dendrites around amyloid plaques (Brendza *et al.*2003; Tsai *et al.*2004a). Treatment of cultured neurons with fibrillar Abeta results in an increase of Tau phosphorylation, leading to a loss of microtubule-binding capacity and accumulation of Tau in the somatodendritic compartment (Busciglio *et al.*1995). Moreover, apolipoprotein E4 (ApoE4), the major genetic risk factor for AD, leads to excess amyloid build up in the brain before AD symptoms arise. Thus, Abeta deposition precedes clinical AD (Polvikoski *et al.*1995).

Advances in the understanding of AD pathogenesis provide strong support for a modified version of the amyloid hypothesis, which is now often referred to as the Abeta cascade hypothesis. The basic tenant of this modified hypothesis is that an intermediate misfolded form of Abeta, neither a soluble monomer nor a mature aggregated polymer but an oligomeric species, triggers a complex pathological cascade leading to neurodegeneration (Barghorn *et al.*2005; Kokubo *et al.*2005).

The relationship between APP, axonal transport and aberrant Abeta processing is not as easy as for Tau. Axonopathy and transport deficit can be detected long before extracellular Abeta deposition in AD patients and in a mutant APP mouse model (Stokin *et al.*2005). Overexpression of human APP695 also impairs specific components of axonal transport in Drosophila and mice (Gunawardena & Goldstein 2001; Salehi *et al.*2006). In mice, this leads to degeneration of basal forebrain cholinergic neurons (BFCN). Conversely, Abeta itself might impair axonal transport, possibly as oligomeric Abeta_42_ in microtubule-associated endosomal vesicles (Hiruma *et al.*2003; Maloney *et al.*2005; Takahashi *et al.*2004). In conclusion, impairment of axonal transport might be a cause or an effect of aberrant Abeta production or, in some cases, result from APP overexpression (Adalbert *et al.*2007).

The latter two theories point out the relevance of axonal transport for proper neuronal function. Finally, ApoE4, the major risk factor for sporadic AD, may directly disrupt the cytoskeleton and hence impair axonal transport also (Mahley *et al.*2006). Here, we give some insights into how neurotrophins may be the actors allowing to link between cholinergic degeneration, amyloid and Tau pathologies and axonal transport.

## Neurotrophins: the NGF family

**Figure 1:**
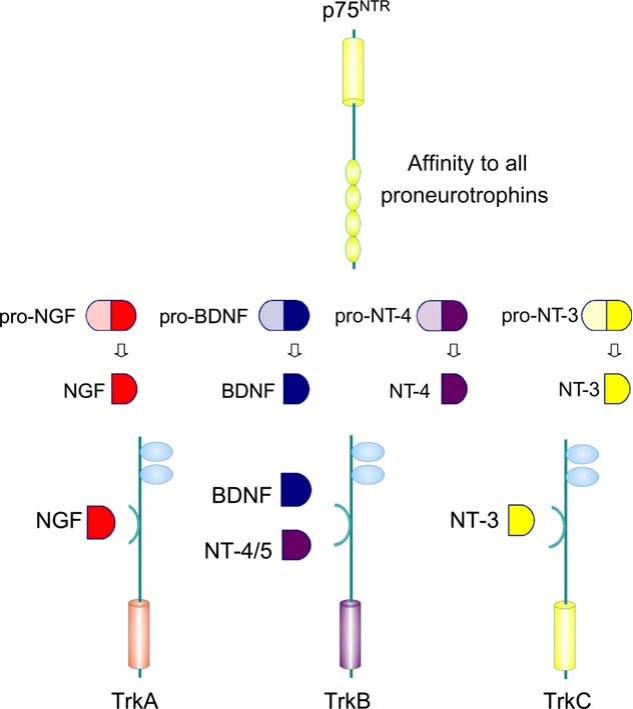
The neurotrophins and their receptors.

The most prominent members of the mammalian neurotrophin family are nerve growth factor (NGF), brain-derived neurotrophic factor (BDNF), neurotrophin-3 (NT-3) and neurotrophin-4/5 (NT-4/5). They activate various cell signaling pathways by activating two types of membrane-bound receptors, Trk (actually ‘tropomyosin-related kinase’ but recently ‘tyrosine receptor kinase’ is also used: TrkA, TrkB and TrkC) and p75^NTR^. These neurotrophins are synthesized as proneurotrophins that all bind to the p75^NTR^. In their active cleaved form, each neurotrophin selectively activates one of three types of Trk receptors (Fig. 1), NGF activates TrkA, NT-3 activates TrkC, while both BDNF and NT-4 activate TrkB receptors (Patapoutian & Reichardt 2001). The role of proneurotrophins and neurotrophins appears to be contradictory: while neurotrophins maintain survival and function, to certain neuronal populations, proneurotrophins trigger cell death through p75^NTR^ (Friedman 2000).

These neurotrophic factors (NTF) are small, versatile proteins that maintain neuronal survival, axonal guidance, cell morphology and play key roles in cognition and memory formation. During embryonic development, NTF are essential for the proper architecture and function of the brain. Knockout mice for NGF, BDNF and NT-3 are all fatal and exhibit severe neural defects. Subsequent to neuronal injury and lesions (like cerebral ischemia), NTFs are upregulated and are involved in healing and neurogenesis.

Axonal transport processes are essential for proper NTF signaling. Nerve growth factor, for example, is synthesized far away from its site of action. Vesicles containing NTF and their relevant receptors are shipped along neuronal projections throughout the brain as summarized in Table 1. However, most neurodegenerative dementias are linked to failures in axonal transport and – not surprisingly – the majority of them are associated with impaired regulation and imbalance of NTF.

**Table 1:** Axonal transport and function of NFT

Neurotrophin	Site of synthesis	Transported to (neuronal population)	Transport	Function
NGF	Neocortex	ChAT-positive neurons of the nbM	Retrograde	Survival and maintenance of cholinergic, sensory and sympathetic neurons
	Hippocampus	ChAT-positive neurons of the MS, VDB, HDB and sum		
BDNF	Frontal cortex	Parietal, cingulate, infralimbic, orbital, perilimbic and occipital cortices, contralateral frontal cortex, nbM, hypothalamus, locus coeruleus, thalamus and HDB	Retrograde	Survival and maintenance (of dopaminergic neurons), synaptic plasticity (long-term potentiation, neuronal firing rate, neurotransmitter release and synaptic transmission) and metabolic effects
	Occipital cortex	Retrosplenial, perirhinal, temporal, entorhinal and frontal cortices, Raphe nucleus, VDB (HDB), thalamus, lateral geniculate nucleus and hypothalamus		
	Hippocampus	Ipsi- and contralateral subfields of hippocampus, MS, sum and VDB (HDB)		
	Entorhinal cortex	Subiculum, CA1 and CA3 hippocampal subfields, amygdala, MS and VDB		
	Amygdala	Temporal, parietal and occipital, entorhinal, cingulate, infralimbic, insular piriform and perirhinal cortices, thalamus, dorsal Raphe, (pre)subiculum and CA1 subfields of hippocampus, medulla, HDB, hypothalamus, nbM and substantia nigra pars compacta		
	Striatum	Frontoparietal cortex, TH-positive neurons of the substantia nigra, Raphe and thalamus		
	Amygdala	Stria terminalis	Anterograde	
	Neocortex	Striatum		
	Dentate gyrus	CA3 subfield of hippocampus (through mossy fibers)		
	Pons	Amygdala		
NT-3	Hippocampus	MS, VDB, thalamus and sum of hypothalamus	Retrograde	Survival and maintenance

HDB, horizontal limb of diagonal band of Broca; MS, medial septum; sum, supramammilary nucleus; TH, tyrosine hydroxylase; VDB, vertical limb of diagonal band of Broca.

## Neurotrophins and their receptors in AD

### Nerve growth factor

Pro-NGF is the predominant form of NGF in the human and rodent brain, whereas mature NGF can be hardly detected. In AD, pro-NGF is increased in frontal and occipital cortex (Crutcher *et al.*1993; Fahnestock *et al.*2001; Hellweg *et al.*1998; Peng *et al.*2004) and in hippocampus (Hock *et al.*2000a; Narisawa-Saito *et al.*1996; Scott *et al.*1995), while a loss is observed in the basal forebrain (Mufson *et al.*1995; Scott *et al.*1995). The amount of NGF messenger RNA (mRNA) is not altered in AD (Fahnestock *et al.*1996; Goedert *et al.*1986, 1989; Jette *et al.*1994). A decrease of retrograde transport could explain this observation, leading to an accumulation of NGF at the sites of its production (hippocampus and neocortical areas) and a deficiency at the NGF transport terminus, the BFCN.

In the absence of NGF, cholinergic neurons show cell shrinkage, reduction in fiber density and downregulation of transmitter-associated enzymes [e.g. choline-acetyl transferase (ChAT) and AChE], resulting in a decrease of cholinergic transmission (Svendsen *et al.*1991). In parallel, rats show a decrease in ChAT and TrkA mRNA after fimbria transection that can be restored by NGF treatment (Venero *et al.*1994).

In AD, a reduction of ChAT and AChE activity and BFCN size and number was observed (Arendt *et al.*1983; Kasa *et al.*1997; Loy *et al.*1990; Perry *et al.*1992; Treanor *et al.*1991), implicating a severe cholinergic degeneration. Therefore, the classical AD therapy was treatment with AChE inhibitors that enhance neuronal transmission by increasing the availability of ACh at the receptors. This effect is beneficial to stabilize cognitive function and to improve or stabilize many behavioral symptoms of AD at a steady level during a 1-year period of treatment (Giacobini 2003; Wynn & Cummings 2004). Currently, there is an ongoing gene therapy trial using NGF-grafted autologous fibroblasts that were injected into the basal nucleus of Meynert (nbM) (Tuszynski *et al.*2005) with the aim to rescue the BFCN of AD patients.

Moreover, a loss of the NGF receptor TrkA was found in the basal forebrain (Boissiere *et al.*1997; Chu *et al.*2001; Ginsberg *et al.*2006a; Mufson *et al.*1997, 2000; Salehi *et al.*1996) and in the cortex (Counts *et al.*2004; Hock *et al.*1998; Savaskan *et al.*2000) of AD brains.

The reports about p75^NTR^ in AD are not that clear: one study observed an upregulation of p75^NTR^ in hippocampal tangle-bearing neurons (Hu *et al.*2002), another unchanged cortical levels without referring to tangle pathology (Counts *et al.*2004; Hock *et al.*1998; Perry *et al.*1993), while in nbM, p75^NTR^ appears to be unchanged (Ginsberg *et al.*2006b; Mufson *et al.*2003) or decreased (Kerwin *et al.*1992; Mufson *et al.*2002; Salehi *et al.*2000). Moreover, during aging, a switch from TrkA to p75^NTR^ occurs, resulting in increased amyloidogenic processing of APP (Costantini *et al.*2005, 2006).

However, there is another interesting link between NGF and APP: neuronal cell cultures upregulate APP expression when treated with NGF (Mobley *et al.*1988; Robakis *et al.*1991; Villa *et al.*2001). In fact, it was shown that NGF acts on the APP promoter mediated by p75^NTR^ and upregulates APP transcription and the secretion of secreted amyloid precursor protein (sAPP) (Ge & Lahiri 2002; Rossner *et al.*1998), although intraparenchymal NGF delivery did not significantly increase Abeta deposition in monkeys (Tuszynski *et al.*1998).

However, neuronal cell models secrete more NGF and downregulate TrkA and p75^NTR^ when treated with Abeta or H_2_O_2_ (Olivieri *et al.*2002). Excitingly, the receptor levels of p75^NTR^ increase initially, indicating that vesicular stores of p75^NTR^ appear to fuse to the plasma membrane. The toxicity of Abeta is mediated by p75^NTR^ through p75-like apoptosis-inducing death domain (PLAIDD), inhibitory G protein, C-Jun N-terminal kinases (JNK), reduced nicotinamide adenine dinucleotide phosphate oxidase and caspase-9 and caspase-3 (Costantini *et al.*2005; Hashimoto *et al.*2004; Tsukamoto *et al.*2003). Moreover, NGF potentiates Abeta toxicity shifting the half maximal effective concentration (EC_50_) from 0.1 μm to 1 pm (Yankner *et al.*1990).

The interaction between NGF and Tau in AD or tauopathies is less clear: NGF-induced neuronal differentiation of the neuroblastoma cell line pheochromocytoma celline-12 (PC-12) exhibits an increase in Tau promoter activity and subsequently elevated Tau protein levels (Sadot *et al.*1996). In addition, NGF also regulates Tau phosphorylation: stimulation of differentiated PC-12 with NGF caused a dephosphorylation of Tau proteins (Fisher *et al.*1996), and NGF deprivation induced hyperphosphorylation of Tau (Nuydens *et al.*1997; Shelton & Johnson 2001). Moreover, NGF induces ubiquitination of Tau in cultured cells (Babu *et al.*2005), indicating that NGF may regulate Tau protein levels by inducing proteasomal degradation of Tau.

According to the hypothesis that NGF deprivation is one of the factors involved in the etiology of sporadic forms of AD, a mouse model (AD11 anti-NGF mice) had been developed, based on the expression of transgenic antibodies neutralizing NGF. The model is characterized by a progressive neurodegenerative phenotype defined by the deposition of amyloid peptide, by intracellular neurofibrillary tangles and by a marked cholinergic depletion (Capsoni *et al.*2002). In addition, spatial memory and neocortical long-term potentiation are impaired in AD11 mice at an age corresponding to early neurodegenerative stage characterized by the first observed decrease in the number of BFCNs without overt cortical neurodegeneration. Acute pharmacological treatment with NGF, ACh or an AChE inhibitor can rescue these symptoms (De Rosa *et al.*2005; Origlia *et al.*2006).

Nerve growth factor expression is regulated by cholinergic innervation from the basal forebrain (da Penha Berzaghi *et al.*1993) and by hippocampal *N*-methyl-d-aspartate (NMDA) receptors (Thoenen *et al.*1991) to maintain the normal levels. Kainic acid induces an increase of NGF transcription that can be blocked by benzodiazepine. In that light, it is exciting that treatment with the NMDA antagonist memantine had no effect on the regulation of NGF in a lesion model (Lang *et al.*2004).

But NGF is not found in neuronal cells only in the AD brain. Astrocytes and microglia show high levels of NGF (Siegel & Chauhan 2000). Inflammatory signals (cytokines and complement factors) as well as Abeta_25-35_ are potent stimulators of human microglial NGF synthesis (Heese *et al.*1998). In addition, hippocampal astrocytes incubated with Abeta upregulate NGF expression and its release to the culture medium. Moreover, these astrocytes display increased Tau phosphorylation and reduce the survival of cocultured hippocampal neurons (Saez *et al.*2006).

### Brain-derived neurotrophic factor

Brain-derived neurotrophic factor regulates synaptic plasticity and thus plays a key role in memory formation and storage (Hellweg & Jockers-Scherubl 1994). Therefore, the involvement of BDNF in dementia has been discussed extensively. In that light, it is not surprising that mRNA (Connor *et al.*1997; Garzon *et al.*2002; Holsinger *et al.*2000; Phillips *et al.*1991) and protein (Ferrer *et al.*1999; Hock *et al.*2000a; Michalski & Fahnestock 2003; Peng *et al.*2005) levels of BDNF are decreased in hippocampus and neocortex of AD brains (for review, see Murer *et al.*2001; Siegel & Chauhan 2000).

Three out of six transcripts, which code for BDNF, are downregulated (Garzon *et al.*2002). Excitingly, two of these are controlled by a cyclic adenosine 5′-phosphate response element-binding protein (CREB) responsive promoter. However, CREB deregulation appears to be involved in the pathogenesis of AD (Yamada *et al.*1997; Yamamoto-Sasaki *et al.*1999).

Not only is BDNF diminished, but also its full-length receptor TrkB is analogously reduced in hippocampus and frontal cortex in AD (Allen *et al.*1999; Ferrer *et al.*1999). The fate of TrkB in BFCN remains to be elucidated: there are two studies reporting a decrease (Ginsberg *et al.*2006b; Salehi *et al.*1996) and another indicating no changes (Boissiere *et al.*1997).

Alzheimer’s disease is tightly associated to neuroimmunological processes (Heneka & O’Banion 2007). Regulation of TrkB in glia differs from that in neurons. Upregulation of truncated TrkB receptors has been found in association with senile plaques (Allen *et al.*1999; Connor *et al.*1996; Ferrer *et al.*1999). In addition, increase of full-length TrkB was observed in glial-like cells in hippocampus and increase of BDNF in dystrophic neurons surrounding senile plaques (Ferrer *et al.*1999). This was confirmed in the APP23 mouse model and shown to be related to neuronal sprouting (Burbach *et al.*2004).

Only a few studies do not support the loss of BDNF or TrkB in AD (Durany *et al.*2000; Hock *et al.*1998; Savaskan *et al.*2000). However, most data above refer to mRNA or protein levels in neurons. In activated glia, the regulation of BDNF and truncated TrkB is induced. One of these studies reporting an increase of BDNF in AD was performed using an enzyme-linked immunosorbent assay and so, no data were available concerning plaque densities. Possibly, this study population presented a rather high plaque concentration in hippocampus, resulting in high glial BDNF reactivity. Other brain areas that were examined with the same method showed the reported loss of BDNF (Durany *et al.*2000).

The role of single-nucleotide polymorphism in AD is still a matter of debate. Polymorphism of the BDNF has been implicated with higher risk for AD. Especially for non-ApoE4 carriers and in specific ethnic groups, this effect is well documented (Akatsu *et al.*2006; Desai *et al.*2005; Forero *et al.*2006; Huang *et al.*2007; Kunugi *et al.*2001; Matsushita *et al.*2005; Nishimura *et al.*2005; Olin *et al.*2005; Riemenschneider *et al.*2002; [Bibr b184][Bibr b185]).

Other studies observed no association with BDNF polymorphism (Bian *et al.*2005; Chuu *et al.*2006; Combarros *et al.*2004; Lee *et al.*2005b; Li *et al.*2005; Nacmias *et al.*2004; Saarela *et al.*2006; Vepsalainen *et al.*2005); so, it remains to be elucidated whether or not this effect is mainly restricted to the Asian population. Compared with wild-type populations, the polymorphisms C270T and V66M appear to be overrepresented in AD. The first is located in a noncoding region and is responsible for the transcription of BDNF mRNA transcript 4, the latter affects BDNF transport and secretion.

But there are more interactions of AD and BDNF: a specific loss of BDNF was found in tangle-bearing neurons (Ferrer *et al.*1999; Murer *et al.*1999), and BDNF dephosphorylates Tau including the most crucial sites for microtubule binding through TrkB activation and a PI3-kinase/Akt-dependent mechanism in a cellular model (Elliott *et al.*2005), implicating a direct Tau–BDNF interaction.

A very interesting link is the fact that during aging and in AD, Tau pathology starts in the entorhinal cortex and proceeds along the retrograde transport pathways of BDNF to the subiculum and the CA1 subfield and then to the basal forebrain, amygdala and finally to several cortical regions.

The interaction of BDNF and the APP promoter is still not that clear as it is for NGF: one study denies an upregulation of APP mRNA after BDNF treatment (Rossner *et al.*1998), while other reports state upregulation in SH-SY5Y cells mediated by MAPK/Ras and PI3/Akt (Ruiz-Leon & Pascual 2004) or promoter activity in PC12 cells (Ge & Lahiri 2002). In addition, the latter group showed in a neurologic disorder associated with increased cerebral BDNF-enhanced plasma levels of full-length APP and nonamyloidogenic APP (Sokol *et al.*2006).

Oligomeric Abeta but not fibrillar Abeta_42_ decreases specifically phospho-CREB and the BDNF transcripts IV and V in differentiated SH-SY5Y neuroblastoma cells (Garzon & Fahnestock 2007), confirming the data that sublethal doses of Abeta without specifying the aggregation form downregulate BDNF and CREB in cultured cortical neurons (Tong *et al.*2001b, 2004). In contrast, another study found out that differentiated SH-SY5Y cells treated with Abeta upregulate full-length TrkB and BDNF and downregulate truncated TrkB. This effect can be reversed with an antioxidant, indicating that this is mediated by oxidative stress (Olivieri *et al.*2003).

Another link combining BDNF and AD pathogenesis is BDNF as a regulator of GSK-3β: BDNF increases the phosphorylation of S9-GSK-3β, which turns the kinase activity off (Mai *et al.*2002).

Physical and cognitive activity and housing mice in an enriched environment increases BDNF and other neurotrophin levels (Chen & Russo-Neustadt 2005; Tong *et al.*2001a). However, the effect of this on amyloid pathology in murine APP transgenic models remains to be elucidated: two studies report a decrease of amyloid burden (Ambree *et al.*2006; Lazarov *et al.*2005), one reports no changes (Wolf *et al.*2006) although demonstrating a raise of BDNF and NT3 and finally, one study observes even an increase of amyloid pathology (Jankowsky *et al.*2003). Curiously, a decrease of BDNF regulation was observed during training on spatial navigation in the APP23 mouse, whereas wild-type mice show an increase (Hellweg *et al.*2006). Nevertheless, it should be kept in mind that enriched housing, cognitive training and wheel running act also on many factors other than BDNF only, so the outcome can be additionally related to aspects other than BDNF.

BDNF regulation is maintained through cholinergic innervation and through NMDA receptors (da Penha Berzaghi *et al.*1993; Thoenen *et al.*1991). The maintenance of normal BDNF mRNA levels appears to be mediated predominantly by NMDA receptors, whereas the increases in BDNF above normal levels are mediated by non-NMDA receptors. Interestingly, the NMDA receptor antagonist memantine used as treatment against AD increases the levels of BDNF and TrkB in rats (Marvanova *et al.*2001).

### NT-3 and NT-4/5

Neurotrophin-3 mRNA and protein levels are unchanged in the AD brain (Durany *et al.*2000; Hock *et al.*1998, 2000a; Murase *et al.*1994; Phillips *et al.*1991), besides a minor reduction of NT-3 in the motor cortex of AD patients, a brain structure often preserved in AD (Narisawa-Saito *et al.*1996). In addition, cerebrospinal fluid (CSF) levels of NT-3 are not changed either (Hock *et al.*2000b).

A possible association of missense mutation (G63E) of the NT-3 gene with AD was found in a Japanese cohort. This association was more prominent among those who did not carry the ApoE4 allele than those who carried the ApoE4 allele (Kunugi *et al.*1998).

PC12 cells show increased APP promoter activity subsequent to NT-3 treatment; however, compared with NGF, this effect is rather mild (Ge & Lahiri 2002). In primary cultures of cortical neurons, NT-3 protects neurons against Abeta toxicity by limiting caspase-8, caspase-9 and caspase-3 cleavage. This neuroprotective effect of NT-3 was concomitant to an increased level of Akt phosphorylation and mediated through phosphoinositide 3-kinase (PI-3K). Moreover, NT-3 induces an upregulation of neuronal apoptosis inhibitory protein-1 expression in neurons that promote the inhibition of Abeta-induced neuronal apoptosis (Lesne 2005). In contrast to NGF, NT-3 does not induce apoptosis through p75^NTR^ in neuroblastoma cells (Kuner & Hertel 1998). Finally, NT-3 prevents the degeneration of noradrenergic neurons of the locus coeruleus in a lesion model that resembles the pattern of cell loss found in AD (Arenas & Persson 1994).

Protein levels of NT-4/5 appear to be slightly decreased in hippocampus and cerebellum, but mRNA levels are not altered in the parietal cortex of AD patients (Hock *et al.*1998, 2000a).

Neurotrophin-4/5 induces Tau dephosphorylation through TrkB, while NT-3 mediated by TrkC does not show the same effect (Elliott & Ginzburg 2006). Therefore, one can speculate that a lack of endogenous TrkB or impaired BDNF/NT-4/5 signaling may lead to Tau hyperphosphorylation.

Curiously, differentiated SH-SY5Y cells treated with Abeta upregulate NT-4/5 (Olivieri *et al.*2003).

### Fibroblast growth factor-2

Although not belonging to the neurotrophin family, fibroblast growth factor-2 (FGF-2 or formally known as basic FGF) shares many similarities with the classical neurotrophins. Fibroblast growth factor-2 is important in neuronal development and neuroprotection after neuronal lesions (Cheng & Mattson 1992). Interestingly, it regulates BDNF and *vice versa*(Johnson-Farley *et al.*2007; Kiprianova *et al.*2004; Soto *et al.*2006).

Increased levels and enhanced binding of FGF-2 were detected in senile plaques and neurofibrillary tangles in AD brains (Gomez-Pinilla *et al.*1990; Kato *et al.*1991; Siedlak *et al.*1991; Stieber *et al.*1996) and in CSF from AD patients (Hanneken *et al.*1995). Moreover, it was shown that FGF-2 increases the neuritic involvement of plaques (Cummings *et al.*1993). Immunoreactivity of the FGF receptor-1 that binds FGF-1 and FGF-2 is increased in AD in reactive astrocytes surrounding senile plaques (Ferrer & Marti 1998; Takami *et al.*1998).

Incubation of neuronal cultures with FGF-2 results in increased Tau phosphorylation (Burack & Halpain 1996) by increasing the levels of the Tau kinase GSK-3β and Tau itself (Butt & Dinsdale 2005; Jin *et al.*2003; Tatebayashi *et al.*1999, 2003, 2004).

Fibroblast growth factor-2 acts also on the APP promoter mediated by p75^NTR^, upregulates APP transcription and the secretion of sAPP (Ge & Lahiri 2002; Rossner *et al.*1998; Villa *et al.*2001), but somewhat weaker than NGF does. Glial cells exposed to Abeta produce more FGF-2 (Araujo & Cotman 1992; Pike *et al.*1994). Double transgenic mice overexpressing APP and FGF-2 display a higher mortality than mice expressing APP alone (Carlson *et al.*1997). But FGF-2 expression does not act by increasing the amyloidogenic processing of APP to Abeta peptides. In contrast, FGF-2 inhibits Abeta-induced neurotoxicity mediated by p75^NTR^ in neuronal cultures (Costantini *et al.*2005; Hashimoto *et al.*2004; Tsukamoto *et al.*2003).

## Conclusions

Neurotrophic factors are key regulators not only for development, maintenance and survival but also for cognition, formation and storage of memory. In AD, NTF are dysregulated and because of impaired axonal transport, unevenly distributed.

**Figure 2:**
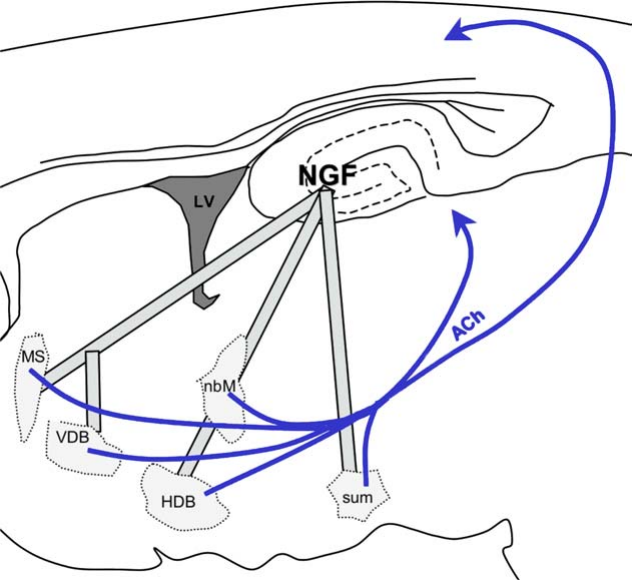
Retrograde transport of NGF from the hippocampus to the basal forebrain Nerve growth factor maintains survival and function of BFCN. Cholinergic projections (in blue) innervate the neocortex and hippocampus and regulate transcription of BDNF. HDB, horizontal limb of diagonal band of Broca; LV, lateral ventricle; MS, medial septum; sum, supramammilary nucleus; VDB, vertical limb of diagonal band of Broca.

In aging, Tau proteins are becoming increasingly hyperphosphorylated, leading to the formation of neurofibrillary tangles in the transentorhinal and entorhinal cortex. As not only Tau but also APP and ApoE4 play a key role in axonal transport (Adalbert *et al.*2007), it would not be surprising that even at this early stage, deficits in transport processes can occur. Fascinatingly, the progression of neurofibrillary pathology in aging and in AD is identical to the retrograde transport pathways of BDNF in this neuroanatomical region. Under physiological conditions, BDNF is produced in the entorhinal cortex and shipped from here through the CA3 to the CA1–subiculum area, basal forebrain and amygdala, the next stations of neurofibrillary degeneration through the AD brain. One cannot exclude impaired transport of BDNF or downregulation of BDNF in tangle-bearing neurons in the aged brain, both leading to deficits in BDNF levels associated with possibly subclinical insufficiency in cognition and memory. Moreover, Tau pathology is the first visible occurrence of brain aging, but APP or low doses of Abeta or ApoE4 pathology may also influence the axonal transport of NTF at this stage. Furthermore, once the neurofibrillary pathology reaches the basal forebrain (occasionally already in Braak stage I), impaired retrograde transport of NGF could be the consequence, leading to an accumulation of NGF where it is synthesized (hippocampus and neocortex) and to a loss of NGF in the basal forebrain (Fig. 2). The well-known degeneration of BFCN in AD could be the outcome of this scenario. Additionally, cholinergic degeneration leads to a decrease in cholinergic innervation from fibers projecting from the basal forebrain to hippocampus and neocortex and thereby, to a decline of basal levels of BDNF expression with all its possible consequence on Tau phosphorylation. But what is more, NGF accumulation in the target regions may upregulate APP, but also may lead to increased signaling of pro-NGF through p75^NTR^, which is increasingly expressed in the aged brain, and thus mediates cell death. Tau could function upstream to Abeta to modify APP transport. Blocking APP transport *in vivo*increases Abeta generation and deposition. Some studies implicate that tau is required for Abeta toxicity, suggesting that tau lies downstream of Abeta.

Not surprisingly, all major proteins involved with AD pathology (APP and Tau) or risk for sporadic AD (ApoE4) are associated somehow with axonal transport. However, using this knowledge for the development of therapy is not as simple.

The most important concern regarding a future therapy with NTF is the mode of delivery. Being small proteins with roughly no penetration of the blood–brain barrier, new avenues for therapy need to be found. An ongoing gene therapy focusing on NGF-grafted autologous fibroblasts that are implanted into the basal forebrain of AD patients predicts a slower progression of the dementia, some cognitive improvement and sprouting of axons on the site of injection (Tuszynski *et al.*2005). Nevertheless, this therapy includes brain surgery and gene therapy and does not appear to be suitable as prophylactic cheap treatment for millions of aging people worldwide. Probably, NTF signaling is more likely a target for AD therapy than the NTFs themselves.

More data and support are needed to elucidate the mechanisms of NTF imbalance and dysregulation in AD. With this knowledge, we will be able to target pathways upstream NTF deregulation or deficits in axonal transport, thus starting the therapy before pathological imbalance of NTF occurs. This could include inhibitors of Tau kinases to avoid pathological Tau hyperphosphorylation that interferes with axonal transport processes and BDNF regulation. Unfortunately, chronic GSK-3β inhibition with lithium ions, which are used in therapy against bipolar disorder, appears not to have the predicted protective effect against AD (Bauer *et al.*2003; Chuang 2004; Manji *et al.*1999), although it had been shown to regulate endogenous BDNF and NGF levels (Frey *et al.*2006a, b).

Nevertheless, the potentials of neurofibrillary tangles (NFT) or drugs that act on their distribution or signaling should be considered carefully as future AD therapy.
